# 5-Aminolevulinic acid regulates the immune response in LPS-stimulated RAW 264.7 macrophages

**DOI:** 10.1186/s12865-018-0277-5

**Published:** 2018-12-19

**Authors:** Yuta Sugiyama, Yukari Hiraiwa, Yuichiro Hagiya, Motowo Nakajima, Tohru Tanaka, Shun-ichiro Ogura

**Affiliations:** 1 School of Life Science and Technology, Tokyo Institute of Technology, Yokohama, Kanagawa Japan; 2 SBI Pharma CO., LTD., Roppongi, Tokyo, 106-6020 Japan

**Keywords:** 5-Aminolevulinic acid, Macrophage, Heme oxygenase-1, LPS, Anti inflammation

## Abstract

**Background:**

Macrophages are crucial players in a variety of inflammatory responses to environmental cues. However, it has been widely reported that macrophages cause chronic inflammation and are involved in a variety of diseases, such as obesity, diabetes, metabolic syndrome, and cancer. In this study, we report the suppressive effect of 5-aminolevulinic acid (ALA), via the HO-1-related system, on the immune response of the LPS-stimulated mouse macrophage cell line RAW264.7.

**Results:**

RAW264.7 cells were treated with LPS with or without ALA, and proinflammatory mediator expression levels and phagocytic ability were assessed. ALA treatment resulted in the attenuation of iNOS and NO expression and the downregulation of proinflammatory cytokines (TNF-α, cyclooxygenase2, IL-1β, IL-6). In addition, ALA treatment did not affect the phagocytic ability of macrophages. To our knowledge, this study is the first to investigate the effect of ALA on macrophage function. Our findings suggest that ALA may have high potential as a novel anti-inflammatory agent.

**Conclusions:**

In the present study, we showed that exogenous addition of ALA induces HO-1 and leads to the downregulation of NO and some proinflammatory cytokines. These findings support ALA as a promising anti-inflammatory agent.

## Background

Recent research has revealed a variety of macrophage roles and functions, and macrophages have a very important role in maintaining homeostasis and a normal physiological condition by adjusting various biological activities. When the elaborate balance of macrophage activity collapses, macrophages can cause various diseases [1]. For example, a number of recent investigations have indicated the important relationship between the particular steps of colorectal cancer development and inflammation due to obesity, which is mediated by proinflammatory cytokines (e.g., interleukin-6 (IL-6)) and tumor necrosis factor-α (TNF-α) secreted by macrophages [2, 3]. Therefore, to avoid lifestyle-related diseases caused by macrophages, it is important to prevent abnormal activation and to keep macrophages within their proper range of activity. Lipopolysaccharide (LPS) is a component of the outer membrane of Gram-negative bacteria. Since LPS is widely used in studies of inflammation and chronic inflammation can be modeled by administration of LPS in vivo [4, 5], we used LPS to study inflammation in an in vitro model.

Heme oxygenase-1 (HO-1) is a well-known antioxidant that catalyzes the degradation of heme [6]. Heme digestion implemented by HO-1 leads to the production of biliverdin, ferrous iron, and carbon monoxide. Biliverdin is reduced promptly to bilirubin by biliverdin reductase. It is generally assumed that these heme-related catabolites have antioxidant activity [7]. For example, carbon monoxide mediates potent anti-inflammatory effects. Otterbein et al. (2000) revealed that carbon monoxide at low concentrations inhibited the expression of the lipopolysaccharide (LPS)-induced proinflammatory cytokines TNF-α, interleukin-1β (IL-1β), and others [8].

5-Aminolevulinic acid (ALA) is a natural amino acid and a precursor in the porphyrin synthesis pathway leading to heme [9]. The first and normally rate-limiting step of heme synthesis is the mitochondrial enzyme 5-aminolevulinic acid synthase (ALAS). Thus, exogenous ALA addition leads to the upregulation of heme synthesis and heme-related enzymes. In addition, it is well known that ALA exposure results in HO-1 induction. In particular, Nishio (2014) reported that the addition of ALA to a macrophage cell line (RAW 264 cells) increases heme, which inactivates Bach1, and that HO-1 expression is induced via activation of MAPK [10]. However, the functions of macrophage cells under HO-1 induction by ALA are not well understood.

In this study, we investigated the effect of ALA on macrophage function. To this end, we utilized the LPS-stimulated mouse macrophage cell line RAW264.7, and evaluated the effect of ALA on inflammatory cytokine expression levels and macrophage phagocytic ability.

## Results

### HO-1 protein expression analysis of ALA-treated RAW264.7 macrophages

**Fig. 1:**
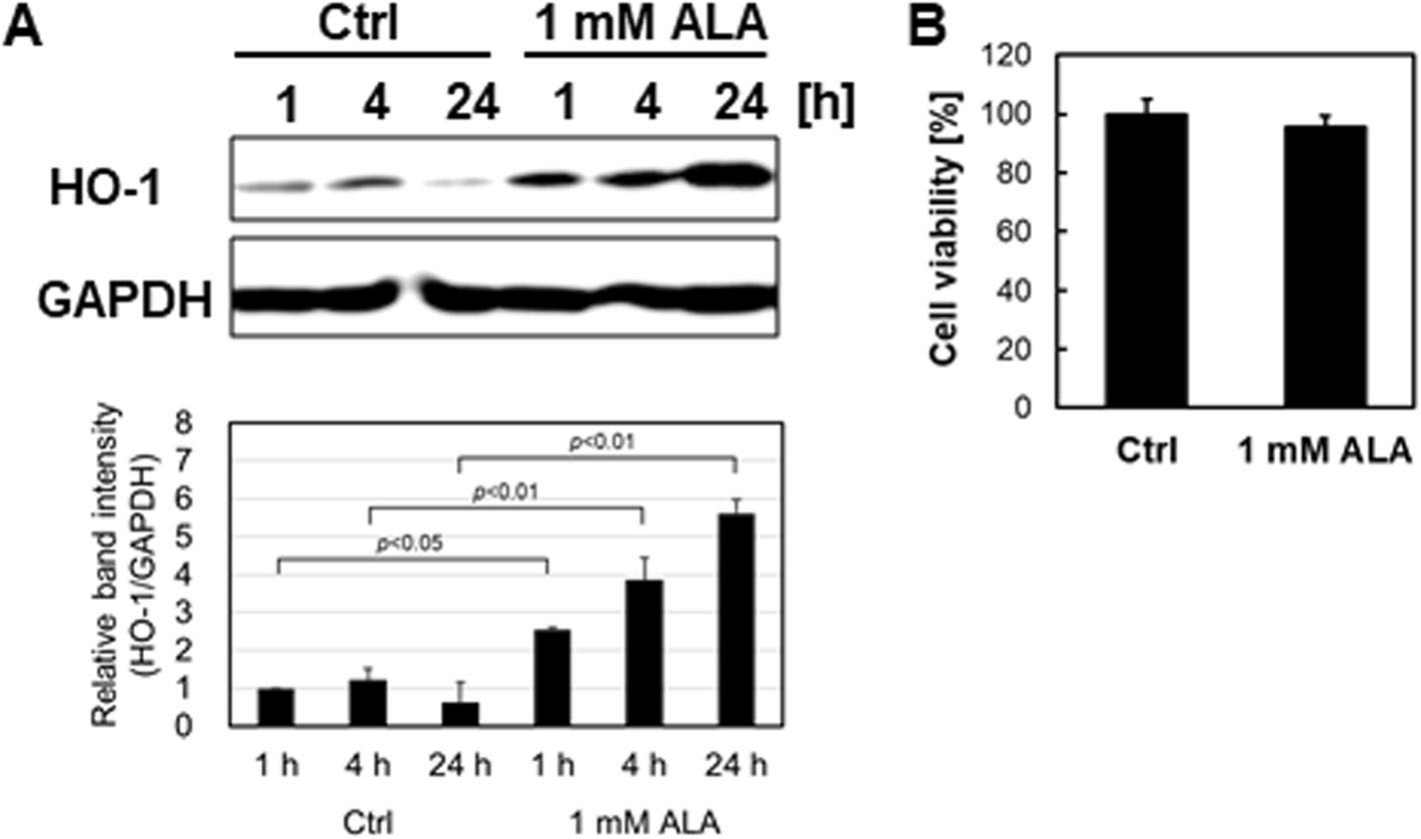
ALA induced HO-1 protein expression level without reducing cell viability in RAW264.7 macrophage. **a** RAW264.7 cells were incubated with or without 1 mM of ALA for 24 h. After treatment, HO-1 protein expression level was analyzed by the Western blotting method. Upper panel, representative Western blot bands. Lower panel, summarized bar graph shows band intensity presented as ratio of HO-1 over GAPDH. Values are means ± SD, *n* = 2. **b** The viability of RAW264.7 cells were tested by MTT assay after 24-h incubation in medium containing 1 mM of ALA. Values are means ± SD, *n* = 3, *p* values are shown in figures

We first examined the effect of ALA on the RAW264.7 macrophage cell line by western blot analysis of HO-1 and MTT viability assays (Fig. 1). Cells were seeded and incubated with medium containing 1 mM ALA. After 24 h of incubation, cells were used for each assessment. HO-1 protein expression levels of the 1 mM ALA-added group were significantly upregulated compared with those of the control group in a time-dependent manner (Fig. 1a). These results are consistent with previous studies [10]. No decrease in cell viability was observed in 1 mM ALA (Fig. 1b). These results suggest that ALA addition induces HO-1 protein expression without noticeable cell damage in RAW264.7 cells.

### iNOS and NO expression analysis of RAW264.7 macrophages under LPS stimulation with ALA

Since we evaluated the effect of ALA alone on HO-1, we investigated the effect of ALA on the activity of the macrophage cell RAW264.7. Nitric oxide (NO), a free radical produced by nitric oxide synthase (NOS), has been shown to have a number of important biological functions, including tumor cell killing and host defense against intracellular pathogens. iNOS is generally not present in inactive cells but is induced by various stimuli such as LPS [11]. Therefore, iNOS and NO expression analysis of RAW264.7 macrophages under LPS stimulation with ALA was performed to assess the effect of ALA on the immune response.

**Fig. 2:**
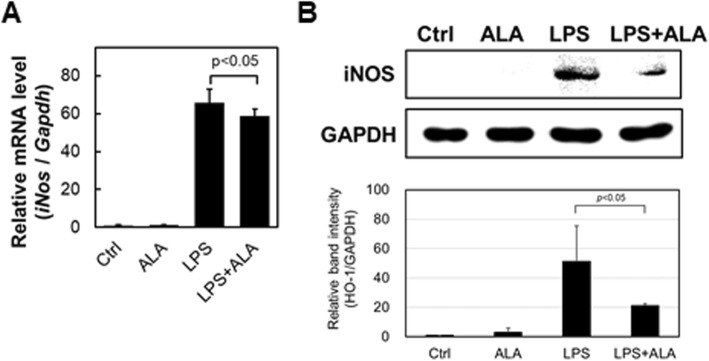
ALA significantly reduced LPS-induced iNOS mRNA and protein expression. RAW264.7 was incubated in 1 μg/mL LPS condition with or without 1 mM ALA. After incubation, **a** iNOS mRNA levels were analyzed by Real-time PCR (Values are means ± SD, *n* = 3) and **b** iNOS protein levels were analyzed by Western blotting (Upper panel, representative Western blot bands. Lower panel, summarized bar graph shows band intensity presented as ratio of iNOS over GAPDH. Values are means ± SD, *n* = 2, *p* values are shown in figures)

RAW264.7 cells were cultured in 1 μg/mL of LPS with or without 1 mM ALA for 24 h. After culture, mRNA and protein expression levels of iNOS were assessed by quantitative real-time PCR and western blot analysis, respectively (Fig. 2). iNOS mRNA expression levels were clearly upregulated under LPS treatment; there was no significant effect on iNOS expression by ALA. However, LPS-stimulated iNOS mRNA expression with ALA was significantly lower than that without ALA (*p* < 0.05). Similarly, iNOS protein expression levels were increased by LPS stimulation and were decreased by ALA treatment. These results therefore confirmed that iNOS was induced by LPS stimulation, which is similar to other reports suggesting that ALA reduced iNOS induction by LPS [12].

**Fig. 3:**
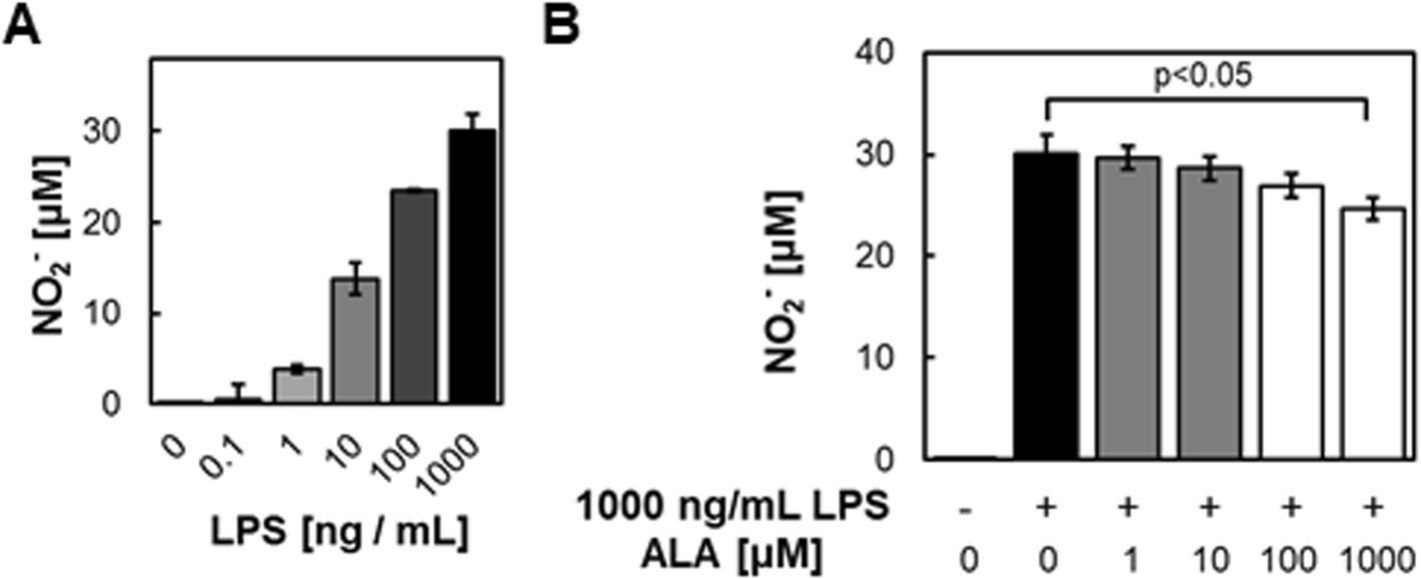
ALA-treatment significantly reduced NO_2_^−^ expression in RAW264.7 macrophages stimulated by LPS. NO_2_^−^ expression levels of RAW264.7 were tested after incubation with several conditions of (**a**) LPS or (**b**) ALA with 1000 ng/mL LPS. Values are means ± SD, *n* = 3, *p* values are shown in figures

Next, NO expression levels of RAW264.7 cells cultured 24 h with each concentration of LPS and ALA were measured by the Griess assay (Fig. 3). First, NO expression levels were tested under the LPS-alone condition. NO was increased in an LPS dose-dependent manner (Fig. 3a). Second, the fluctuation of NO levels with ALA under 1000 ng/mL LPS was measured. A significant reduction of NO was observed in an ALA dose-dependent manner (Fig. 3b). These data indicated that ALA has the ability to downregulate mRNA and protein expression of iNOS, which followed NO synthesis in RAW264.7 cells stimulated by LPS. Since several reports have suggested the relationship of HO-1 and iNOS to NO production, it was suggested that ALA-induced HO-1 may cause the downregulation of iNOS and NO expression [13, 14].

### Proinflammatory cytokine expression analysis of RAW264.7 macrophages under LPS stimulation with ALA

**Fig. 4:**
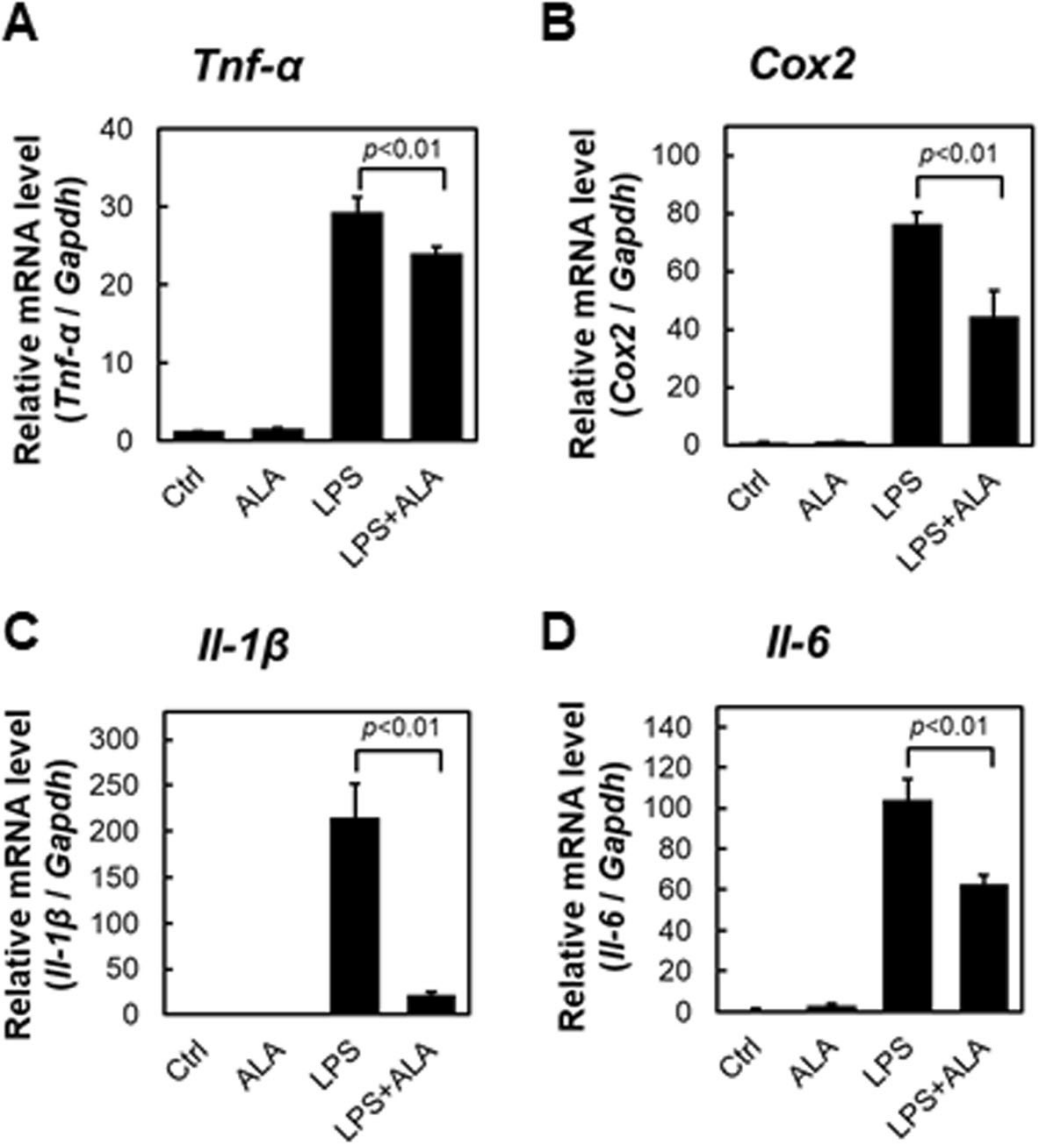
ALA-treatment shown significant inhibitory effect on proinflammatory cytokines. **a** TNF-α, **b** COX2, **c** IL-1β and **d** IL-6 mRNA expression levels of RAW264.7 were analyzed after 1 mL ALA treatment and/or 1 μg/mL LPS. Values are means ± SD, *n* = 3, *p* values are shown in figures

Since ALA showed a decreasing effect on iNOS and NO, we investigated other proinflammatory cytokines known to be induced by LPS stimulation. After LPS stimulation in 24-h culture with or without ALA, the mRNA expression levels of TNF-α, cyclooxygenase2 (COX2), IL-1β, and IL-6 were assessed by real-time PCR analysis (Fig. 4). The mRNA levels of each of the proinflammatory cytokines were upregulated by LPS treatment. In contrast, all of the LPS-inducible cytokines were significantly reduced with ALA addition compared with the LPS-alone condition. These results are consistent with Otterbein et al. (2000) and they suggested that carbon monoxide, which is the by-product of heme degradation by HO-1, inhibits the expression of LPS-induced proinflammatory cytokines. Thus, our data indicated that the addition of ALA leads to HO-1 expression and a downregulative effect on proinflammatory mediators.

### Phagocytic ability assays of RAW264.7 macrophages under LPS stimulation with ALA

**Fig. 5:**
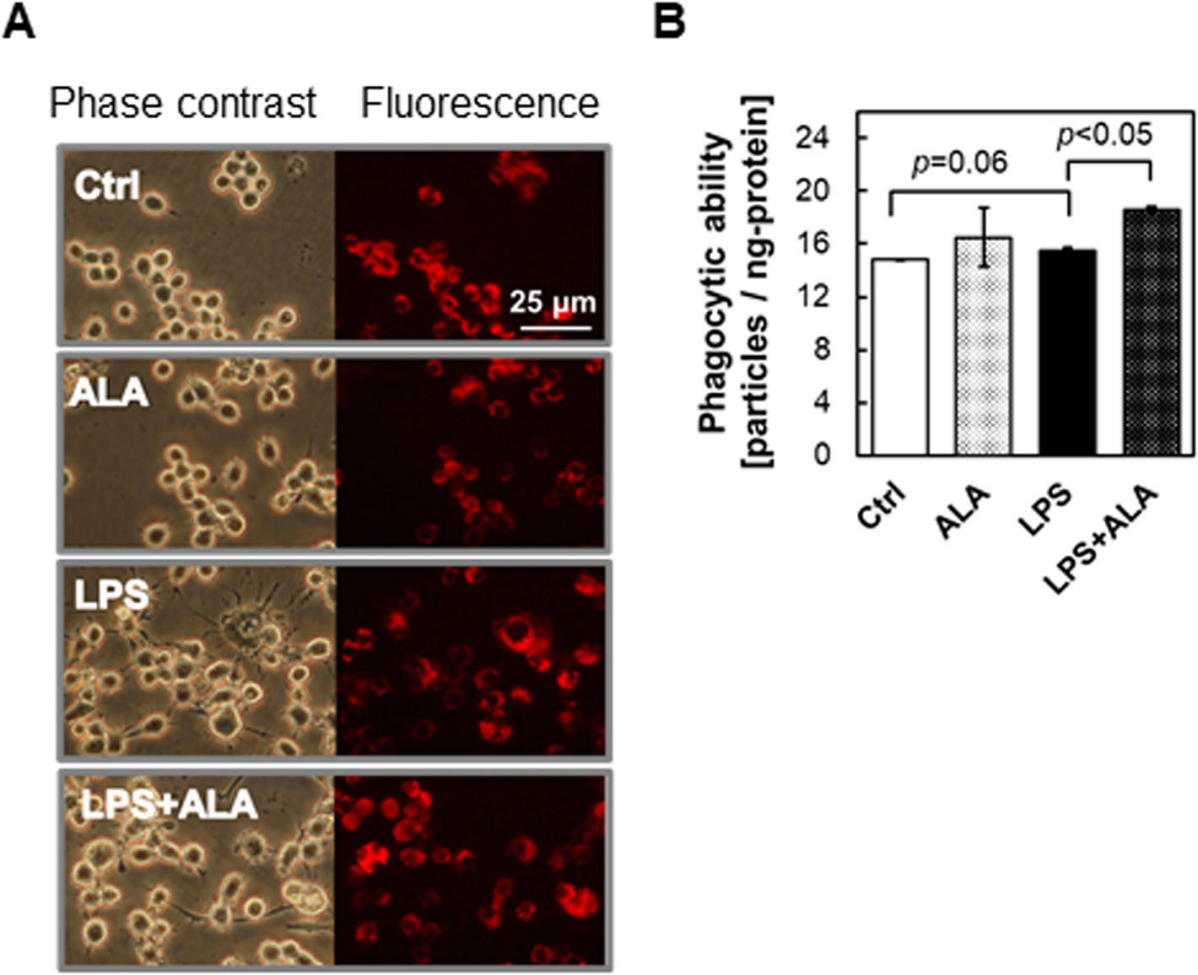
Phagocytotic ability was more strongly increased by co-administration of LPS and ALA than by LPS alone. Phagocytotic ability was estimated using fluorescence beads. RAW264.7 was treated with 1 μg/mL LPS and/or 1 mM ALA for 24 h and incubated for 5 h in culture medium containing 1 × 10^8^ particles of fluorescence beads. **a** Fluorescence microscopy observation was performed (left, phase contrast; right, fluorescence). **b** Fluorescence beads uptake was assessed as phagocytotic ability by using fluorescence spectrophotometer (Ex. 535 nm, Em. 570 nm). Values are means ± SD, *n* = 3, *p* values are shown in figure

In general, phagocytosis is a characteristic feature of macrophages. We tested the effect of ALA on the phagocytic ability of macrophages because it was revealed that ALA has a downregulative effect on proinflammatory cytokines. To examine the phagocytic ability of RAW264.7 cells, we used fluorescence beads. After pretreatment with 1 μg/mL LPS with or without 1 mM ALA for 24 h, RAW264.7 cells were cultured in a medium containing 1 × 10^8^ particles of fluorescence beads for 5 h. Cells were then observed by fluorescence microscopy, and digital images were obtained. Figure 5a shows the intracellular beads in red fluorescence. Figure 5b shows the relative particle intake normalized by cellular protein as phagocytic ability. LPS stimuli induced the upregulation of phagocytic ability (*p* < 0.05). Moreover, the co-incubation of LPS and ALA resulted in significantly higher particle intakes (*p* < 0.01 compared with the LPS-alone condition). These data show that ALA alone has no influence on phagocytic ability. Although our finding show that ALA exhibits a downregulative effect on proinflammatory cytokines, they also show that ALA significantly upregulated phagocytic ability with LPS stimulation.

## Discussion

In this study, we evaluated the functions of ALA-induced expression of HO-1 by macrophage cells by ALA under LPS treatment. First, we showed that ALA addition induces HO-1 protein expression without noticeable cell damage. Second, we revealed that ALA causes changes in several proinflammatory factors induced by LPS stimulation. In particular, ALA treatment induced the attenuation of iNOS and NO expression and the downregulation of proinflammatory cytokines (TNF-α, COX2, IL-1β, and IL-6), with no reductive effect of phagocytic ability, were shown. These data indicate that ALA, as a HO-1 inducer, has promise as a novel anti-inflammatory agent.

HO-1 is the first and rate-limiting enzyme in the action of degrade of heme into carbon monoxide, ferritin, and biliverdin. Accumulating evidence suggests that carbon monoxide and bilirubin, produced from biliverdin by the enzyme biliverdin reductase, are potent anti-inflammatory mediators [6]. It is generally assumed that this HO-1 and a bi-product-related system may act as a crucial regulator in inflammatory processes, and regulates the balance between proinflammatory and anti-inflammatory mediators.

As an immune response to LPS stimulation, macrophages upregulate their production of various inflammatory cytokines. Indeed, there are several reports on the relationship between HO-1 and the LPS-inducible reaction in macrophages. For example, it has been suggested that HO-1 expression is induced via depletion of intracellular glutathione by LPS-induced ROS [15]. Otterbein et al. (2000) have shown that carbon monoxide produced by HO-1 inhibits the expression of LPS-induced proinflammatory cytokines such as TNF-α and IL-1β [8]. They suggest that the effect of HO-1 is based on the mechanism of p38 MAPK inhibition. In addition, carbon monoxide can bind to the iron atom of heme in heme proteins, such as NOS, and modulate their function [16]. Thus, carbon monoxide may weaken the activity of iNOS by combining with heme. On the other hand, bilirubin is generally well known as a potent reducer that can scavenge radical compounds. Since NO produced by iNOS in the immune response is a radical, bilirubin reduces NO, thus suppressing a series of NO reactions [17]. As a broad reaction by several kinds of radicals or immune-responsible factors may occur against immune stimulation, it can be said that bilirubin has an important role in arresting these reactions [18, 19]. To our knowledge, Fe^2+^ does not have an anti-inflammatory role. Rather, Fe^2+^ has a cytotoxic effect via the Fenton reaction [20]. Thus, we consider that Fe^2+^ did not have a key function as an anti-inflammatory agent of ALA in our study. Berberat et al. reported that the ferritin heavy chain induced by Fe^2+^ protects endothelial cells from apoptosis induced by a variety of stimuli, such as ischemia-reperfusion injury in vitro and in vivo [21]. This finding may offer an anti-oxidative role of Fe^2+^ as the downstream molecule of HO-1. From the above consideration, we hypothesize the following mechanism of the anti-inflammatory effect of ALA. ALA may upregulate heme synthesis, which in turn increases HO-1, which then acts to degrade heme. At the same time, the catabolites of heme increase and show a suppressive effect on iNOS and other proinflammatory cytokines.

Macrophage cells have been shown to contribute to inflammation-related diseases through the secretion of proinflammatory cytokines. As many researchers have reported, the HO-1-related system of macrophages may be a therapeutic target for chronic inflammation [22–24]. For example, Hualin (2012) revealed that hemin upregulated HO-1 expression in rat alveolar macrophages, and that pretreatment with hemin inhibited LPS-induced NO production, developed arginase activity, and enhanced phagocytotic ability via the p38 MAPK pathway in these cells [25]. In addition, Oh et al. showed that HO-1 is induced by the addition of H_2_S [26]. They found that H_2_S suppressed the activation of NF-κB by LPS, which in turn then suppressed iNOS expression and NO production. They also showed that knock down of HO-1 with siRNA cancels the effect of suppression of iNOS expression and NO production. More interestingly, they suggested that CO is a particularly important factor since the same effect as that by adding H_2_S is also obtained by adding CO alone, which is a product of HO-1. We observed HO-1 upregulation (Fig. 1) and attenuation of iNOS and NO expression by ALA exposure (Figs. 2 and 3); therefore, we suggest that a similar mechanism is involved.

In the classical classification, there are two types of macrophages: M1 and M2. The features of M1 macrophages are glycolysis and the secretion of proinflammatory cytokines. On the other hand, M2 macrophages show higher oxidative metabolism and anti-inflammatory cytokine production, such as IL-10. Vats (2006) revealed that the metabolic shift by overexpression of PGC1β attenuates macrophage-mediated inflammation (e.g., suppression of IL-6 expression) [27]. On the other hand, our previous study demonstrated that ALA has an upregulative effect on aerobic respiration based on the induction of the mitochondrial respiratory chain complex IV, a heme protein enzyme named as cytochrome *c* oxidase [28, 29]. We consider that the mechanism of this phenomenon is that ALA administration upregulates heme synthesis. This leads to the induction of hemoprotein synthesis, which is followed by the induction of heme protein assembling, as we have previously shown in cytochrome P450, a heme protein that catalyzes the oxidation of lipophilic substrates [30]. The ALA-effect of NO attenuation and the downregulation of proinflammatory cytokines may be caused by not only the HO-1-related system but also a metabolic shift caused by ALA.

ALA is the natural biochemical precursor of porphyrin synthesis and broadly exists in animals and plants. In animal cells, the synthesized porphyrin is converted to heme with iron by ferrochelatase. The rate-limiting enzyme in porphyrin synthesis is ALA synthetase, the role of which is ALA synthesis from glycine and succinyl CoA in mitochondria. Therefore, exogeneous ALA addition causes the upregulation of porphyrin synthesis. Moreover, in cancer cells, ALA administration induces the accumulation of fluorescent porphyrins [9]. Thus, ALA is a widely used compound for tumor photodynamic diagnosis. For example, the W. Stummer group reported that there are no concerns regarding the toxicological safety of fluorescence-guided surgery with 5-aminolevulinic acid in phase III clinical trials [31].

## Conclusions

In this study, we showed that exogenous addition of ALA, the precursor of heme, induces HO-1 and leads to the downregulation of NO and some proinflammatory cytokines. To our knowledge, this study is the first to investigate the effects of ALA on macrophage function. These findings support ALA as a promising anti-inflammatory agent. Further studies are needed to comprehensively examine the effect of ALA on macrophages.

## Methods

### Biochemicals

The substrate ALA hydrochloride was purchased from Cosmo Oil Co., Ltd. (Tokyo, Japan). RPMI-1640 medium and Antibiotic-Antimycotic solution (ABAM, Penicillin-Streptomycin-Amphotericin B mixture) were obtained from Nacalai Tesque (Kyoto, Japan). Fetal bovine serum (FBS) was purchased from Invitrogen (Carlsbad, CA, USA). The MTT reagent was purchased from Sigma-Aldrich (St. Louis, MO, USA). All other chemicals used were of analytical grade.

### Cell culture and viability assays

The mouse macrophage cell line RAW264.7 was purchased from RIKEN Cell Bank (Ibaraki, Japan). The cells were grown in DMEM, supplemented with 10% FBS and ABAM, and incubated at 37 °C in an incubator with a controlled humidified atmosphere containing 5% CO_2_. Cell viability was measured by the MTT assay as described previously [32].

### Western blot analysis

Western blot analysis was performed as previously described with some modifications [33]. For immunoblot analysis, samples were first treated with the SDS-PAGE sample buffer solution containing 10% (*v*/v) 2-mercaptoethanol. Thereafter, sample proteins were electrophoretically separated by 15% polyacrylamide gels and then electroblotted onto a polyvinylidene fluoride membrane (Millipore, Bedford, MA). The membrane was incubated in blocking solution containing 5% (*w*/*v*) skim milk in TTBS [20 mM Tris-HCl (pH 7.4), 150 mM NaCl, 0.05% (v/v) Tween 20] at 4 °C overnight. We used an antibody specific to HO-1-1 (1:200, mouse monoclonal, ab13248, Abcam, Cambridge, UK) [34], iNOS (1:500, inducible nitric oxide synthase, mouse monoclonal, sc-7271, Santa Cruz Biotechnology, Inc., TX, USA) [35] or GAPDH (1:1000, mouse monoclonal, 05–50,118, American Research Products, Inc., Belmont, MA, USA) [36] as the primary antibody. For the secondary antibody, we used anti-mouse IgG horseradish peroxidase (HRP)-conjugated antibody (Cell Signaling Technology, Inc., Beverly, MA, USA) at 1:3000 dilution. HRP-dependent luminescence was developed with Western Lightning Chemiluminescent Reagent Plus (PerkinElmer Life and Analytical Sciences, Inc., Waltham, MA, USA) and detected with a Lumino Imaging Analyzer ImageQuant LAS 4000 mini (GE Healthcare UK, Amersham Place, England). The intensity of chemiluminescence was determined with an ImageQuantTM TL Analysis Toolbox (GE Healthcare UK, Amersham Place, England).

### Quantitative real-time PCR analysis using SYBR green assays

Total RNA was isolated using a NucleoSpin® RNA II (Macherey-Nagel, Düren, Mannheim, Germany) kit according to the manufacturer’s instructions. The concentration and quality of RNA were analyzed using a UV-Vis spectrophotometer (Shimadzu). Subsequently, cDNA was synthesized from total RNA using a PrimeScript RT reagent kit with a gDNA Eraser (TaKaRa Bio, Otsu, Japan) according to the manufacturer’s instructions. The expressions of iNOS, COX2 (cyclooxygenase2), TNF-α, IL-1β, IL-6, and GAPDH mRNAs were determined using a Thermal Cycler Dice® Real-Time System Single (TaKaRa Bio, Shiga, Japan) with a SYBR Premix Ex Taq (TaKaRa Bio). Primers are shown in Table 1.

**Table 1:** Sequences of primers used in real-time PCR

iNOS	forward	5’-CCTCCTCCACCCTACCAAGT-3’
	reverse	5’-CACCCAAAGTGCTTCAGTCA-3’
COX2	forward	5’-AGGAGACATCCTGATCCTGGT-3’
	reverse	5’-GTTCAGCCTGGCAAGTCTTT-3’
TNF-α	forward	5’-GTGGAACTGGCAGAAGAGGC-3’
	reverse	5’-AGACAGAAGAGCGTGGTGGC-3’
IL-1β	forward	5’-CCTCGTGCTGTCGGACCCAT-3’
	reverse	5’-CAGGCTTGTGCTCTGCTTGTGA-3’
IL-6	forward	5’-CCGGAGAGGAGACTTCACAG-3’
	reverse	5’-CAGAATTGCCATTGCACAAC-3’
GAPDH	forward	5’-TGTGTCCGTCGTGGATCTGA-3’
	reverse	5’-TTGCTGTTGAAGTCGCAGGAG-3’

The amplification conditions included 30 s at 95 °C; 50 cycles at 95 °C for 5 s and 60 °C for 60 s each; dissociation for 15 s at 95 °C and 30 s at 60 °C; and then 15 s at 95 °C on a Thermal Cycler Dice Real-Time System. Thermal Cycler Dice Real-Time System analysis software (TaKaRa, Shiga, Japan) was used for data analysis. The Ct values (cycle threshold) were calculated using the crossing-point method, and the genes expression levels were measured by comparison with a standard curve. The expression levels of target genes were normalized to those of GAPDH.

### Measurement of nitric oxide (NO)

Nitrite, a stable metabolite of NO, was measured with a Griess Reagent System (Promega, Madison, WI, USA) according to the manufacturer’s instructions.

### Phagocytic ability assays

Phagocytosis assays were performed using Fluoresbrite™ Yellow Orange (YO) Carboxylate Microspheres (18720–10, Polysciences, Inc., Warrington, PA, USA) according to the manufacturer’s instructions. Briefly, 5 × 10^6^ cells were inoculated in a 3 cm dish and incubated with LPS and ALA for 24 h following medium change. After the incubation, medium was changed to 2 mL of fresh medium without FBS and 20 μL of 1 × 10^8^ particles/mL of Fluoresbrite® Microparticles solution was added. After 5 h of incubation, cells were washed three times with PBS. Cells were then observed with a fluorescence microscope (CKX41, Olympus, Japan) equipped with a digital camera (E620, Olympus), and digital images were obtained. Fluoresbrite® Microparticle fluorescence was measured at excitation and emission wavelengths of 535 nm and 570 nm, respectively. To determine the intracellular particle number, the macrophages were collected by a cell scraper and resuspended in PBS. The Fluorescence intensity was measured (excitation and emission wavelengths of 465 nm and 550 nm, respectively) and compared to a standard curve obtained from fluorescence particles alone in PBS. After measurement, cells were lysed by 0.1 M NaOH and protein concentration was measured by Bradford protein assay.

### Statistical analysis

Data are expressed as means ± standard deviation in two or three independent experiments. Statistical significances were analyzed using the Tukey-Kramer’s test with an α level of 0.05. Statistical analyses were performed using a software, JMP® 13 (SAS Institute Inc., Cary, NC, USA).

## Abbreviations

ABAM: Antibiotic-Antimycotic solution
ALA: 5-Aminolevulinic acid
ALAS: 5-Aminolevulinic acid synthase
COX2: Cyclooxygenase2
FBS: Fetal bovine serum
HO-1: Heme oxygenase-1
IL-1β: Interleukin-1β
iNOS: Inducible nitric oxide synthase
LPS: Lipopolysaccharide
NO: Nitric oxide
TNF-α: Tumor necrosis factor-α
